# Functional Graphene Coatings in Electrochemical Energy Technology—Beyond Corrosion Protection †

**DOI:** 10.3390/molecules30071436

**Published:** 2025-03-24

**Authors:** Qunting Qu, Lijun Fu, Lili Liu, Veniamin Kondratiev, Rudolf Holze

**Affiliations:** 1 College of Energy, Soochow University, Suzhou 215006, China; qtqu@suda.edu.cn; 2 State Key Laboratory of Materials-Oriented Chemical Engineering, School of Energy Science and Engineering, Nanjing Tech University, Nanjing 211816, China; 3 Institute of Chemistry, Saint Petersburg State University, 199034 St. Petersburg, Russia; 4 Chemnitz University of Technology, D-09107 Chemnitz, Germany; 5 Confucius Energy Storage Lab, School of Energy and Environment, Southeast University, Nanjing 210096, China

**Keywords:** graphene, graphene oxide, reduced graphene oxide, few-layer graphene, coating, electrode material, batteries, supercapacitors, fuel cells

## Abstract

Coating the surfaces of active masses and auxiliary components in devices of electrochemical energy technology with graphene and closely related materials has been suggested and experimentally verified in numerous examples. The results in terms of improved performance are promising and suggest the need for further research and technological development. This report provides a complete overview, providing details that are relevant for understanding the way in which these coatings work. Suggestions and directions for further development are indicated.

## 1. Introduction

Graphene (GR) and its chemical relatives, graphene oxide (GO), reduced graphene oxide (rGO), and few-layer graphene (flG), have enjoyed enormous popularity for numerous purposes and applications, inspired by the knowledge of their highly attractive materials properties and many suggestive, perhaps sometimes slightly overoptimistic, research reports. An earlier overview on the use of graphene in electrochemical energy technology (EET) is available [1]. The sometimes encountered (but mostly not stated) assumption that rGO and graphene are the same (presumably based on the assumption that, e.g., reduced copper oxide and copper are the same), as found in, e.g., [2], is not correct, as discussed in [3].

**Figure 1:**
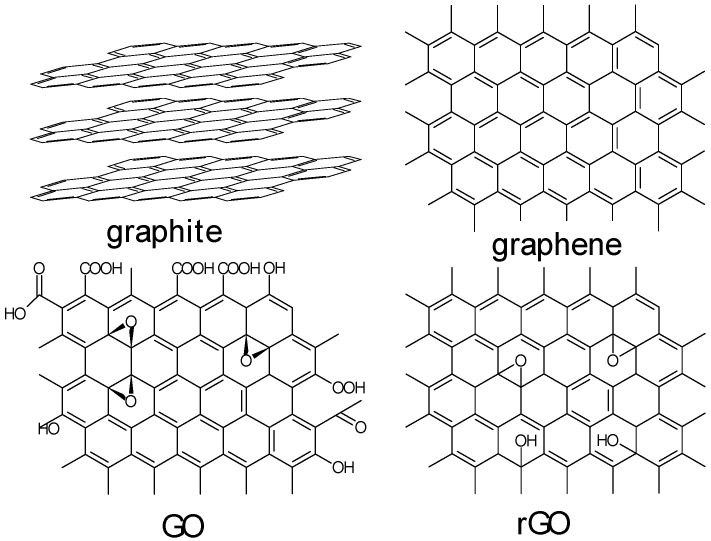
The molecular structures of graphite, graphene, graphene oxide, and reduced graphene oxide.

The salient details of the members of this family of layered carbonaceous 2D materials, such as their molecular structures, are displayed in Figure 1. Graphene as well as flG, GO, and rGO are frequently exploited in different applications due to their tunable optical and electrical properties, very high specific surface area (~2600 m^2^·g^−1^), and catalytical properties with various surface functional groups. Graphene, as a single-atom-thick carbon sheet of hexagonally arranged carbon atoms, is a pioneer member of this family. It can act as a building block for the formation of different distinct carbon-based structures, such as spherical fullerenes, carbon nanotubes, and flat graphite with a typical honeycomb lattice structure.

The essential property for electrochemical applications of such materials to be discussed later is their electronic conductivity (for an overview, see Table 1). However, the reported values vary widely in the literature; thus, only a qualitative comparison is provided. Few-layer graphene sheets have increased electrical conductivity up to ~10^4^ S·m^−1^, which is comparable to that of pristine graphite. In turn, there is a big difference in electrical conductivity between GO and rGO. While GO shows insulating or semi-conducting properties with low conductivity, rGO shows rather high electrical conductivity of up to 10^3^ S·cm^−1^. The introduction of heteroatoms (N, B, S, etc.) into the carbon network leads to additional free electrons or holes in the carbon structure, increasing the electrical conductivity and causing the appearance of new catalytic properties.

Beyond the use of these materials from the graphene family as active electrode masses in, e.g., secondary batteries and supercapacitors [3,12], they can be used as coating materials for active and auxiliary materials. Rather popular is the use of such coatings for corrosion protection, as reviewed elsewhere with respect to the contribution of such coatings toward slower aging in devices and systems of electrochemical energy technology (EET) [13,14]. An experimental transmission electron microscopy (TEM) platform for the study of nanobatteries, including the examination of graphene coatings, has been reported [15].

**Figure 2:**
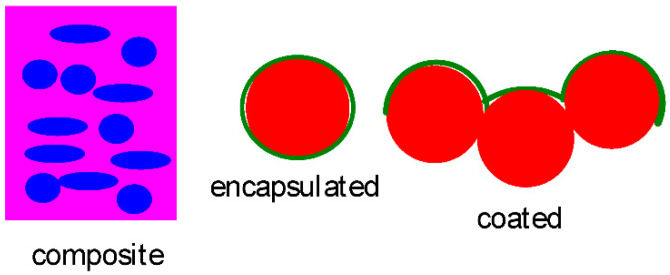
Some illustrations of common terms.

Among the numerous reports on the use of G, GO, rGO, and flG in EET, the majority deal with their use as active masses or as components in mixtures or composites (see, e.g., [3]). A significant minority of these publications describe their use as coatings. Certainly, such coated materials may be called composites, but unfortunately this handling and terminology result only in confusion (for examples, see [16,17,18]). Sometimes terminology oscillates confusingly between composite, embedding, and coating [19]. Reports are included here as long as coating-like effects can be assumed based on the reported evidence, whereas reports wherein the process of deposition of the active mass on some support or current collector is called a coating are not included (see, e.g., [20]). In the absence of any binding, or at least the generally accepted definition of the term “coating” (which seems not necessary when considering common language), it may be appropriate to assume that a coating makes up only a small gravimetric fraction of the material. Cases like ref. [21], with an optimum graphene content of 40 wt%, or with 35.3 at. % carbon content (including graphene) as in [22], may seem to stretch the term coating a bit. Sometimes care in reporting and proper use of terms appear to be less than convincing; although reduced graphene oxide and graphene may be considered from a chemical point of view as being identical, there seem to be significant differences [3]. Accordingly, in the following text, assignment of a coating to any of said materials is based on the provided experimental details, not on confusing terminology (for some illustration see Figure 2). Active masses deposited by layer-by-layer assembly, as in [23], may also be called coated in a slightly misleading way, making them not relevant in the present report. In another report about a layered electrode with active mass particles sandwiched between the current collector and a top graphene coating, the relevance of this report is certainly given [24]. Adding to this confusion is possibly the use of some other terms in the reports presented. A coating is a top layer of some other material applied (coated) either on the electrode or on the material later made into an electrode. A coating might indeed—depending on the method—cover only the apparent surface but not the complete particle. Encapsulation may be considered as a complete “all around” coating, possible only when it is either applied by a suitable method to particles made into an electrode or—less likely to succeed—by some immersive method to the electrode. Depending on the application, the distinction may be relevant or even important.

The present report provides an overview as complete as possible of the reported applications of such coatings, their mode of coating, and the noticed benefits, including the proposed reasons for the improvements. This shall enable the reader to pick a suitable coating, method of applying it, and the expected benefits.

## 2. Applications

In this chapter, the reported applications of coatings with the materials introduced above are presented. To make the collected reports more accessible, they are grouped according to the three main fields of EET and the main components in these systems. In the case of multi-functional coatings, corrosion protection as one task out of several in these reports is covered in a separate review [25]. In case of doubt, the interested reader might wish to consult this source too.

### 2.1. Battery Electrode Coatings

Graphene or reduced graphene oxide coatings have been suggested and tested as the coatings of electrode materials in secondary batteries.

#### 2.1.1. Aqueous Batteries

Coating of a copper electrode for an aqueous copper/aluminum battery with rGO resulted in corrosion protection and generally improved cell performance [26]. Whereas details of the corrosion protection action are provided, the reasons for the improved cell performance are not given.

#### 2.1.2. Nonaqueous Lithium-Ion Batteries

Negative electrode materials

Silicon as a promising (in terms of charge storage capability) material for the negative electrode in lithium-ion batteries suffers from poor electronic conductivity and major volume changes, resulting in mechanical fragmentation during cycling. In addition to the formation of composites (see, e.g., [27]), coatings have been tried. A graphene coating on tremella-like nanostructured silicon resulted in major performance improvements [11]. The volume changes were taken care of by the voids in the tremella-like structure, whereas the poor electronic conductivity was compensated by the coating. Silicon nanotube bundles were coated with graphene, resulting in a five-fold increase in achieved capacity when compared to the uncoated material [28]. The role of a mechanical protection layer was identified as the major role of the coating, providing mechanical stabilization and a stable solid electrolyte interphase (SEI). Graphene coated on porous silicon as an active mass improved the rate capability and storage capacity, and the stability during 100 cycles was also improved significantly when compared to the performance of the bare material [29]. Graphene bonded on an oxidized surface of submicron-size silicon nanoparticles by hydrogen interactions yielded a negative electrode material with remarkable rate capability and mechanical stability [30]. Surface treatment of silicon particles before coating was studied in [31]; thermal surface oxidation in advance of CVD formation of the graphene coating was found to be very advantageous in terms of almost no capacity losses during 300 cycles as compared to a steady decline without such thermal treatment. Using a mixture of CO_2_ and CH_4_ as source for graphene formation instead of a mixture of H_2_ and CH_4_ yielded better performing silicon electrode material [32]. flG coated by CO_2_-enhanced CVD silicon nanoparticles was examined as a negative electrode material [33]. The addition of CO_2_ as a mild oxidant to CH_4_ as a graphene source resulted in a highly conformal coating; the role of CO_2_ was elucidated using DFT calculations. Lithium ion migration in the silicon–graphene system was examined using theoretical tools [34]. Apparently, the coating helped this migration in a way not revealed so far. How this affected intercalation/deintercalation appeared to be beyond the scope of this study. Graphene sheets grown on silicon nanocones provided longer cycling life, better rate capability, higher Coulombic efficiency, and lower electrode polarization [35]. Silicon lithiation-enhanced charge transfer at the silicon–graphene interface was the subject of a first principles study [36]. Silicon particles trapped between a copper current collector and a soft graphene top coating were kept in place by this coating, successfully yielding an electrode with a capacitance that was stable for about 450 cycles [19]. Coating of silicon-polydopamine nanoparticles with graphene improved mechanical cycling stability and capacity by enhancing the electronic conductivity of the material [37]. Further applications of CVD-deposited graphene on nanostructured silicon were collected in [38]. Silicon nanoparticles were encapsulated into rGO bubbles, with the filled bubbles forming a film used as the negative electrode, and the product was sometimes called a composite [39]. The bubble film accommodated volume changes during cycling, stabilized the SEI, and supported ionic and electronic conduction. A stable SEI was also claimed as a major benefit of coating silicon nanoparticles with flG when using an ionic liquid as the electrolyte [40]. Silicon nanoparticles coated with crumpled rGO were examined as a negative electrode material [41]. The crumpled morphology of the coating accommodated the substantial volume changes of the silicon during cycling, contributing to significantly improved performance in terms of increased capacity, stability, and Coulombic efficiency. Silicon nanoparticles were coated with polyaniline and GO, yielding after pyrolysis a material with inner carbon and outer rGO coatings [42]. The dual coating accommodated volume changes during cycling, stabilized the SEI, and kept the silicon nanoparticles from agglomerating during 200 cycles (instead of 50 without the rGO coating). A porous Si-Sn-alloy prepared by high-energy ball milling coated with graphene and subjected to selective etching (which removed one of the alloy constituents) provided stable capacitance during 600 cycles when Si was left and 300 cycles when Sn was left [43]. The improvement was attributed to the voids left by the removal of one alloy constituent, leaving voids that accommodated volume changes during cycling.

In addition to elemental silicon, its monoxide SiO has been suggested as a negative electrode material. Unfortunately, its use faces the same problems as silicon, as already addressed above. Coating with graphene yielded major improvements in remedying these shortcomings [44]. Enhanced performance was also observed after coating SiO_x_ particles with graphene sheets [45]. Similar results were reported elsewhere, with corresponding arguments regarding observed improvements [46,47].

Nanoparticles of Ge were coated with graphene (the title of the report is slightly confusing) [48]. The cycling stability during 90 cycles suggested some effect in suppressing pulverization as well as particle agglomeration.

Graphene coating of hollow nanospheres of SnO_2_ yielded a negative electrode material with attractive and stable performance, which in the absence of data obtained with uncoated material could not be judged by comparison [49]. Coating of composites of SnO_2_ and carbon nanotubes with graphene alleviated the drawbacks (poor electronic conductivity and cycling stability because of volume changes during cycling) of this otherwise attractive negative electrode material [50]. Microspheres of SnS_2_ coated with rGO afforded better performance in terms of higher rate capability and better cycling stability than uncoated SnS_2_ [51]. The improvements were attributed to faster lithium ion diffusion and higher ionic conductivity.

A “composite” of Li_4_Ti_5_O_12_ with 0.5 wt% graphene showed improved performance, which was ascribed to accelerated lithium ion diffusion and ionic conductivity [52]. Given the very low graphene content, the term “coating” used elsewhere in the report appears to be more appropriate. Nanowires of surface-fluorinated Li_4_Ti_5_O_12_ were combined with rGO into a composite for use as a negative electrode in lithium-ion batteries [12]. Elsewhere in the report, the beneficial effects of coating with graphene are discussed; apparently the authors considered both materials, graphene and rGO, to be the same. The addition of rGO resulted in increased capacitance and surface fluorination further helped. The increased electronic conductivity of the composite material and accelerated lithium ion diffusion were attributed to rGO addition. In another study, Li_4_Ti_5_O_12_ coated with graphene or some carbon coating (the report is ambiguous in this detail) with and without nitrogen doping performed best when compared to material coated with whatever nitrogen-free material or not coated at all [53]. The coating—at this stage, the authors seemed to prefer graphene—derived from strong binding between the active material and the coating from DFT considerations, in turn stabilized the electrode/electrolyte interface and reduced the chemical activity of the mass. This may provide a way toward reducing the effects of volume changes (which have been claimed to be absent with so-called zero-strain materials). In addition, electronic conductivity was improved by the coating. These advantages were apparently enhanced by nitrogen doping.

Mesoporous particles of NiFe_2_O_4_ coated with graphene were examined as a negative electrode material for lithium-ion batteries [54]. The increased ionic conductivity and prevention of aggregation during cycling were attributed to the coating. Microrods of Co_3_O_4_ coated with graphene yielded significant improvements in terms of capacity and, to a lesser extent, cycling stability when compared to the performance of pristine material [55]. Nanosheets of Co_3_O_4_ grown on a thin layer of rGO (which was later called a coating) on nickel foam improved both Li^+^ and Na^+^ storage performance by enhancing adhesion to the current collector [56]. Electrophoretic co-deposition of Co_3_O_4_ and graphene nanoplates yielded a binder-free electrode material [57]. In the slightly confused description, the authors oscillated between co-deposition and coating; in any case, with graphene, the reported capacity was about 25% smaller. NiO, as an attractive negative electrode material, gained structural stability and sufficient electronic conductivity by CVD coating with graphene [58]. Coating of cobalt-free LiNiO_2_, suggested as a positive electrode material, suppressed oxygen release at high states of charge during cycling, which in turn caused structural damage and performance degradation [59], with more details reported by these authors elsewhere [60]. Similar results were obtained with LiCoO_2_ [61]. Oxygen release from the lithium-rich positive electrode mass could be suppressed by regulation of redox couples near particle surfaces by coating with fluorinated graphene [62]. Nanotubes of electrospun NiO/Co_3_O_4_ coated with rGO (not graphene as claimed in the title) showed significantly improved performance, which was attributed to increased electronic conductivity and suppression of the negative effects of volume changes by the tubular structure [63]. Microspheres of NiCoMnO_4_ wrapped with graphene by electrostatic self-assembly were examined as an electrode material, with much improved performance in terms of storage capability and stability when compared to the uncoated material [64]. LiNi_0.5_Mn_1.5_O_4_ particles were coated with sulfonated graphene, improving electronic conductivity and accelerating lithium ion transport [65]. Much longer cycling life, greater rate capability, and less growth of the SEI were stated as the noted improvements. Coatings of hollow and solid micropencils of Co_3_V_2_O_8_ were considered in a report—not more [66].

The poor electronic conductivity of FeP and its volume changes during cycling as the negative electrode in lithium-ion batteries were ameliorated with a reduced graphene oxide rGO coating, obtained from a graphene oxide GO solution with subsequent reduction during further chemical treatment [67]. Ultrafine particles of FeSe were embedded in an elastic carbon structure and finally coated with graphene, yielding a stable, mechanical stress-resistant electrode material for lithium-ion and also for sodium-ion batteries [68]. Compared to plain FeSe, the capacity was almost doubled, and it actually improved during 300 cycles.

Coating of graphitic carbon-coated Fe_2_O_3_ contained in conductive carbon nanofibers with onion-shaped graphene layers yielded a stable material for a negative electrode [69]. The graphene coating prevented peeling off of the particles of Fe_2_O_3_ from the carbon nanofibers and increased the electronic conductivity. The electrochemical performance as well as cycling stability of α-Fe_2_O_3_ nanoparticles was improved by coating with graphene [21]. At an optimum graphene content of 40 wt%, the term composite may be more appropriate, as was sometimes used by the authors in the report. Particles of a composite of carbon/Fe_3_O_4_ with a graphene coating acting as a buffering layer showed increased electronic conductivity and mechanical stability [70]. Coating particles of a composite of magnetite (Fe_3_O_4_) and N-doped carbon yielded an electrode material in which the coating prevented volume expansion during cycling, with the associated detrimental effects reducing the stability and cycling lifetime [71]. Hollow nanospheres of Cr_2_O_3_ wrapped in rGO showed significantly increased storage capability [2].

Porous particles of Mn_2_O_3_ were embedded in rGO, whereas elsewhere in the report they were coated with graphene [14]. The authors concluded that rGO provided high electronic conductivity and “prevented large volume expansion”. MnO_2_ pre-intercalated with Na^+^ or ${\mathrm{NH}}_{4}^{+}$ and coated with rGO yielded an electrode material with increased electronic conductivity due to the rGO and better growth morphology because of the intercalated cations [72]. Porous nanospheres of CoMoO_4_ coated with graphene showed higher electronic conductivity and structural stability during cycling as a negative electrode material compared to the uncoated material [73]. Better performance of CVD-deposited graphene coating on nanorods of MnO, suggested as a negative electrode material, than wrapping with chemically exfoliated graphene was reported [74]. The improved cycling stability and rate capability were attributed to better optimization of coating thickness and defect density.

Graphene-encapsulated particles of ZnO for use as a negative electrode material in lithium-ion batteries were prepared by simple spray-drying of a dispersion of GO and a zinc salt solution, followed by heat treatment [75]. The stability of the electrode material and rate capability were significantly improved.

A composite of nanoflowers of CuS and rGO avoided the observed drawbacks of the metal sulfide, i.e., structural deterioration and the “shuttling effect” of the sulfide ions during cycling [76]. Graphene, which actually encapsulated the metal sulfide, strongly adsorbed the soluble polysulfides. MoS_2_ coated with graphene (the material was also called a composite) was studied, but the electrochemical performance was not reported [77].

Layered Ni-Co double hydroxides with an ultrathin conformal graphene coating prepared by electrodeposition (reduction of rGO) showed a major improvement in cycling stability as compared to the uncoated material [78].

A core/shell electrode material of graphene-coated graphite showed enhanced electronic conductivity and in turn better rate capability and capacity when used as a negative electrode material in potassium-ion batteries [79]. The stability and cycling lifetime were also improved.

A hydrophobic graphene coating of lithium metal helped to improve the moisture tolerance of the lithium electrode in lithium-air batteries [80]. A graphene-coated lithium foil used in a lithium metal battery enabled stable cycling for 470 cycles with 76% capacitance retention [81]. Spray-coating of lithium metal yielded a coating with rGO by spontaneous reduction, enabling a dendrite-free metal electrode [82]. Coating a porous copper current collector for the negative electrode in lithium metal batteries with N-doped graphene was proposed as a way to mitigate the effects of volume changes, low cycling efficiency, and safety concerns [83]. Uniform lithium deposition and uniform ion flux were observed. Coating of the lithium metal electrode with multilayer graphene and a Cs^+^ additive in the electrolyte solution improved the electrode performance significantly [84]. The coating separated the metal from the SEI and stabilized the Coulombic efficiency, while the Cs^+^ addition helped to suppress dendrite formation.

Coating of structured copper surfaces with stacked graphene protected the metal electrode from undesired chemical reactions with the electrolyte solution [85].

Positive electrode materials

Pyrophosphate (Li_2_FeP_2_O_7_) is an attractive positive electrode material for lithium-ion batteries. Unfortunately, low electronic conductivity hampers its successful use. A theoretical first principles-based study unraveled the details of the improved ion diffusion induced by the graphene coating of active material particles [86]. Particles of olivine-type LiFePO_4_ were coated with GO, which was subsequently reduced by sintering of the active material [87]. A uniform spherical morphology was observed; at an optimum graphene content of 8 wt.%, stable electrode performance was recorded during 30 (!) cycles. Nanoparticles of LiFePO_4_ were decorated via a catalytic process with graphene and, during the process, the formed graphene sheets formed a cross-linked network in the active mass [88]. The much increased rate capability and cycling stability were attributed to the increased electronic and ionic conductivities of the material. Nickel-doped LiFePO_4_ coated with graphene showed increased conductivity and capacity in comparison to plain LiFePO_4_, which was stable for 20 (!) cycles [89]. Nanostructured LiFePO_4_ co-doped with Nb^5+^ and Ti^4+^ was coated with graphene (actually rGO according to the experimental description) [90]. At a nominal composition of Li_0.99_Nb_0.01_Fe_0.97_Ti_0.03_PO_4_, storage close to the theoretical value was observed. As a major reason for the improvements, the establishment of a 3D conducting network was claimed.

The interface between LiCoO_2_ as the positive electrode and Li_1.5_Al_0.5_Ge_1.5_(PO_4_)_3_ as the solid electrolyte was modified with a layer of graphene [91]. The flexible graphene layer acted as a buffer, reducing the contact resistance between the active mass and solid electrolyte. This generic problem of solid electrolytes has been addressed before [92,93].

LiMn_0.7_Fe_0.3_PO_4_ was coated with electrochemically reduced GO, resulting in higher storage capacity and better stability [94]. Subsequent coating with the chemically reduced GO was less efficient, which was attributed to the formation of a conductive network in the former case. Nanoparticles of (Li_0.893_Fe_0.036_)Co(PO_4_) were combined with graphene into a two-layer sandwich, which appeared to be more particle-like in the report [95]. The presence of graphene in the structure improved the rate capability, and stable cycling during 100 cycles was reported. Hierarchical flower-like particles of Li_2_FeSiO_4_ were coated with graphene (why this was called activation remains unclear) [96]. The obtained material formed a secondary structure with superior structural and thus cycling stability and improved transport properties. A DFT study of graphene-coated iron borate and lithium iron borate was reported [97]. The release of manganese ions by the dissolution of manganese oxide LiMn_2_O_4_ spinel-positive electrodes during cycling was inhibited by coating with single-layer graphene, resulting in greater capacitance stability [98].

Graphene coating of hollow spherical LiNi_0.5_Mn_1.5_O_4_ suppressed structural deformation during cycling and prevented corrosion and the formation of surface defects, resulting in significantly improved performance, with 82.5% capacitance retention after 1000 cycles at the 20 C rate [99]. Micro-nanostructured LiNi_0.5_Mn_1.5_O_4_ embedded in graphene was studied with respect to the question of how a graphene coating benefited this electrode material [100]. Protection against corrosion and stabilization against structural deformation (presumably phase transformation) were found as the main effects. Coating of cerium-doped LiNi_0.5_Co_0.2_Mn_0.3_O_2_ with graphene improved the electrochemical performance in terms of slightly increased capacitance and improved cycling stability [101]. The improved capacitance retention and rate capability were attributed to increased electronic conductivity and protection of the active mass particles against the electrolyte solution. Beneficial effects of a graphene coating were also observed with LiNi_1/3_Mn_1/3_Co_1/3_O_2_ [102]. Particles of nickel-rich LiNi_0.8_Co_0.1_Mn_0.1_O_2_ were coated first with perylene-3,4,9,10-tetracarboxylic dianhydride for better adhesion of the multilayer graphene sheets that were coated thereafter (“snowballing strategy”), yielding a positive electrode material with substantially enhanced storage capability and stability during 100 cycles [103]. Graphene coating (termed here encapsulation) of nickel-rich LiNi_x_Co_y_Mn_1−x−y_O_2_ was suggested as a way to enhance cycling stability and rate capability [104]. These positive effects were attributed to limited access of the electrolyte solution to the active mass and stronger mechanical support mitigating effects of volume change during cycling. In order to achieve a maximum of volumetric as well as gravimetric charge storage capability of an electrode material, the fraction of added auxiliary materials for enhanced electronic conductivity and as a binder must be minimized [105]. Using a Pickering emulsion approach, a graphene coating of nickel-rich LiNi_0.8_Co_0.15_Al_0.05_O_2_ powder was prepared and used as a positive electrode, with only 0.5 wt% content of graphene being equivalent in terms of the conductivity effect to 5 wt% carbon black [106]. In addition to accelerating electron transport surface degradation, surface structural transformation was presumably slowed down. Coating of nickel-rich and magnesium-doped LiNi_0.8_Co_0.1_Mn_0.1_O_2_ with graphene afforded increased structural stability and improved electron and ion conductivities [107]. Coating of another nickel-rich positive electrode material, LiNi_0.84_Co_0.11_Mn_0.05_O_2_, with rGO decorated with V_2_O_5_ was reported [108]. rGO provided increased electronic conductivity whereas V_2_O_5_ facilitates lithium ion intercalation/deintercalation and stabilized the layered structure of the active mass. Improved capacity and better stability as compared to the uncoated material were found. Conformal graphene coating of nanoparticles of a nickel-rich positive electrode material was found to reduce cell impedance, enhance the packing density of the particulate electrode material, and finally increase the cell lifetime four-fold [109]. Conformal coating of nickel-rich NMC532 (LiNi_0.5_Mn_0.3_Co_0.2_O_2_) material slowed down interfacial chemomechanical deterioration [110]. The accelerated electrolyte solution decomposition, in particular at high states of charge, of this “high-voltage” material and chemomechanical strain were mitigated, with associated gains in cycling stability and Coulombic efficiency.

Oxygen loss from lithium-rich Li_2_MnO_3_ can cause structural degradation, which in turn results in low Coulombic efficiency, capacitance fade, and voltage decay. A defective graphene coating can help in stabilizing surface oxygen [111]. The mechanistic details of LiO_2_ extraction from lithium-rich mixed cathode materials Li(Li_0.2_Mn_0.54_Ni_0.13_Co_0.13_)O_2_ were elucidated using in operando X-ray absorption spectroscopy [112]. Further spectroscopic studies of this material by the same authors suggested that graphene coating suppressed the formation of the monoclinic phase, resulting in improved stability [113]. The same active mass was coated with graphene by a spray-drying method, yielding an electrode mass with less solid electrolyte interphase formation and increased electronic conductivity [114].

Coating of phosphorene with graphene improved the stability of this electrode material [115].

The notorious polysulfide shuttle mechanism and the lacking electronic conductivity of sulfur have left this otherwise highly attractive positive electrode material for lithium-ion batteries as an ongoing challenge. Graphene-coated sulfur nanospheres showed 50% capacity retention after 100 cycles [116]. Wrapping, i.e., coating, poly(ethylene glycol)-coated sulfur particles with GO sheets subsequently decorated with carbon black nanoparticles yielded a positive electrode material with a capacitance that was stable for more than 100 cycles [117]. The stability was attributed to the “double coating” accommodating volume changes during cycling and keeping the polysulfides within. A graphene-sulfur composite with 82 wt% sulfur was studied as a positive electrode material [118]. Some graphene acting as a coating provided an electronically conducting matrix and prevented the dissolution of polysulfides. Graphene coating of composites of sulfur with mesoporous carbon yielded an electrode material with the coating accommodating volume changes during cycling, preventing the release of soluble polysulfides and increasing the electronic conductivity of the material [119]. With this material combination, similar improvements were reported elsewhere [120]. In another study, rGO-coated composites of mesoporous carbon and sulfur were examined [121]. The coating slowed down polysulfide diffusion and helped to increase the sulfur content in a way not revealed in the report. Coating of a carbon nanotube-sulfur composite with rGO restrained undesired polysulfide release from the positive electrode [122]. Particles of a composite of multiwalled carbon nanotubes and sulfur were coated (or encapsulated) with rGO, yielding an electrode material with remarkable improved capacity during 200 cycles and slightly better stability than the material without rGO [123]. Somewhat disturbingly, coating showed up only as a keyword together with graphene! This also applies to a report wherein studies of a vesicle-like composite of sulfur and rGO are described [124]. Again, graphene present as nanosheets improved the electronic conductivity and mitigated volume changes of the composite during cycling. A mixture of mesoporous carbon and graphene oxide was melt-infiltrated with sulfur after thermal treatment of the mixture in the presence of ammonia, yielding a mixture with graphene [125]. Whether the graphene acted as a coating (as sometime suggested) or just as a constituent remained unclear; anyway, the composite showed improved performance and capacity retention with current density, suggesting increased electronic conductivity. Polysulfides were trapped in the active mass and nitrogen doping (from the ammonia) enhanced polysulfide adsorption, further inhibiting the shuttle effect. Particles of a composite of carbon nanorods and sulfur coated with graphene showed improved performance due to the coating, which inhibited polysulfide diffusion and enhanced electron and ion transport [126]. Sulfur encapsulated in polypyrrole embedded or sandwiched between rGO showed some increase in capacity when compared with the material without rGO, with the mode of operation of the rGO as stated in preceding examples [127].

The separator inside lithium-sulfur batteries is certainly an auxiliary component; nevertheless, a paper separator coated with multiple CVD-deposited graphene layers inserted as an additional layer between the electrodes of such batteries is possibly better located in this section [128]. The improved performance in terms of increased cycling stability was attributed to unspecified interactions between polysulfide species and graphene. Nanosheets of CoS coated with rGO (elsewhere in the report graphene is mentioned) on a flexible carbon fiber support served as an interlayer in a lithium-sulfur battery, improving polysulfide conversion and Li_2_S decomposition [129]. A coating with B/N-doped rGO and boron nitride nanosheets on the separator of a lithium-sulfur cell enhanced sulfur utilization, inhibited the shuttle effect, and prevented lithium corrosion by lithium polysulfides [130]. Low self-discharge and improved cycling stability of a lithium-sulfur battery was afforded by a sandwich interlayer of VS_2_, carbon nanotubes, and carbon nanofibers coated with graphene [131]. The interlayer suppressed polysulfide shuttling and recovered inactivated sulfur species.

#### 2.1.3. Auxiliary Materials

Coating of current collectors for lithium-ion batteries with flG resulted in lower contact resistance between the current collector and active mass [132]. Simple carbon coating of aluminum foil needed to alleviate, at least in part, poor adhesion, insufficient electrical contact, and localized corrosion initiated by contact with the electrolyte solution was claimed to be too heavy. Instead, coating with a graphene/Ketjen Black mixture or a mixture of graphene micro sheets and graphene was suggested [133]. With the latter coating, lower Ohmic resistance and better adhesion were found. Modification of aluminum foil with carbon black and graphene, used as the current collector for a positive lithium iron phosphate electrode, resulted in an improved rate capability, lower internal cell resistance, and increased cycling stability [134]. The coating increased the effective surface area of the foil, improved mechanical adhesion, and also suppressed corrosion. Graphene modification of copper foil, used as a current collector with Li_4_Ti_5_O_12_ as the negative electrode material in a lithium-ion battery, improved overall performance [135]. The storage capability was increased by 32% in comparison to an uncoated electrode, which was attributed to improved electric contact between the active mass and current collector.

Coating of nickel foam, used as the current collector for a positive electrode, with nitrogen-doped graphene was suggested, and some performance improvements were noticed [136].

Coating of the aluminum surfaces of battery packs for heat dissipation with graphene-copper composites resulted in more even temperature and heat distribution [137]. For overviews on carbon and graphene coatings in thermal management, see [138,139].

#### 2.1.4. Other Metal-Ion Batteries

Graphene-coated particles of FeS_2_ further encapsulated in carbon fiber were examined as the negative electrode for sodium-ion and potassium-ion batteries [140]. High electrochemical reversibility and stability were noticed; for sodium ions, the graphene coating seemed to reduce the diffusion barrier between FeS_2_ and graphene, according to theoretical calculations. The mechanism of capacity fading of micron-sized particles of FeS_2_ was studied [141]. Graphene coating, proper choice of binder, and electrode potential control resulted in high capacity, extended cycling life, and increased rate capability.

Graphene coating of Fe_3_S_4_ was suggested as a way to alleviate the volume changes of the flower-like microstructure of the material and improve its electronic conductivity, resulting in much improved cycling stability [142]. Nitrogen-doped graphene coating of microspheres of FeS_2_ yielded an electrode material for the negative electrode of sodium-ion batteries [143]. Taking into account also theoretical considerations, graphene improved the electronic conductivity of the otherwise poorly conducting sulfide, and it also accelerated sodium ion diffusion kinetics and supported mechanical stabilization. A graphene (actually, according to the report it was rGO) coating on graphite improved its performance as a negative electrode material for potassium-ion batteries [79]. The beneficial effect of the coating, which was evident in the improved cycling stability, was attributed to the buffering capability of the coating, which mitigated the volume changes of the graphite during cycling.

Graphene coating of Co-doped Na_3_V_2−x_Ti_x_(PO_4_)_2_F_3_ yielded a negative electrode material for sodium-ion batteries [144]. The coating improved electrochemical kinetics. NASICON-type NaTi_2_(PO_4_)_3_ conformally coated with graphene yielded a negative electrode material for sodium-ion batteries with high and long-term stable capacity [145]. Somewhat confusingly, the material was called a nanocomposite in the title of the report. Microspheres of Na_4_Fe_3_(PO_4_)_2_(P_2_O_7_), suggested as a positive electrode material for sodium-ion batteries, coated (decorated) with graphene and further embedded in a graphene network showed improved performance, which was attributed to increased electronic conductivity and mechanical stabilization of the material against volume changes during cycling [146].

A noteworthy example of a negative electrode material for sodium-ion batteries without any coating (graphene or other) appears to be SnS [147]. Graphene-encapsulated composites of SnO_2_ and carbon nanotubes showed promising performance as a negative electrode material for sodium-ion batteries [50]. Placing a polypropylene separator coated with polydopamine and multilayer graphene on a sodium metal electrode enabled dendrite-free cycling without significant capacitance loss during 500 cycles [148]. GeP_3_ in a carbon matrix coated with rGO (GeP_3_/C@rGO), suggested as a sodium-storage material, showed higher electronic conductivity and larger surface area than plain GeP_3_ and GeP_3_ coated with graphene [149]. A fourth sample mentioned in the abstract remains a mystery. GeP_3_/C@rGO performed best in terms of capacity, current capability, and long-term stability. rGO protected and restrained the material inside.

Wrinkled graphene sheet coating of hard carbon derived from coal liquefaction residues, as the negative electrode material for sodium-ion batteries, improved performance [150]. The improved performance was attributed to the enhanced electronic conductivity of the active mass by the coating.

The nucleation and growth dynamics of zinc deposition in a zinc metal-free zinc-ion battery were directed by applying a graphene coating on the copper current collector [151]. Further beneficial effects were achieved by coating a zinc electrode with carbon nanofibers and graphene acid (carboxyl-enriched graphene) [152]. The coating was zinc ion selective and prevented zinc corrosion. Coating graphene on the zinc electrode of a zinc-ion battery resulted in significantly improved cyclic reversibility [153]. Although dendrite formation was not inhibited, the coating apparently homogenized the electrode field in front of the electrode, affording even metal deposition.

A FeCo-catalyst for oxygen reduction in a zinc-air battery coated with graphene showed almost four-electron reduction of oxygen [154]. In addition to the remarkable activity stability, methanol tolerance was noted. A bifunctional oxygen electrode catalyst for zinc-air batteries containing B,N-doped graphene nanomesh decorated with Co_3_O_4_ was reported [155]. Why the term coating shows up remains unclear.

A coating of GO on various non-noble metal current collectors prevented electrolyte solution decomposition and their corrosion [156].

Other battery electrode materials

Organic electrode materials suggested as negative electrode materials for aqueous lithium-ion batteries suffer from low electronic conductivity [157]. Coating with graphene significantly improves electrochemical performance.

Conceivable diffusion hindrance due to the steric hindrance established by the graphene coating may be avoided by coating with single walled carbon nanotubes [158]. An active mass of Fe_3_O_4_ was successfully employed as an example.

### 2.2. Supercapacitor Electrode Coatings

The advantageous properties of graphene coating on porous silicon, used as a supercapacitor electrode material, are summarized in [159]; for more similar observations, see [160]. Similar observations were reported for mesoporous silicon oxide wrapped into graphene and used as the negative electrode in a lithium-ion capacitor [161]. The positive effects of graphene coating on the performance of various anthracite-derived carbonaceous materials in lithium-ion capacitors were reported [162]. Particles of electrodeposited Ni(OH)_2_ and Ni coated with graphene in a supergravity field showed increased capacitance and rate capability when compared to the uncoated material [163]. Stability was not examined. Coating of nickel foam with graphene before deposition of ZnCo_2_O_4_, as an active material for a redox supercapacitor, resulted in a substantially different morphology of the formed cobaltate, with significantly increased surface area and porosity and associated improvements in performance [164]. Oxygen vacancy birnessite-type Na_x_MnO_2_, to be used as a positive electrode in a redox supercapacitor, was coated with rGO [165]. The coating increased the electronic conductivity and stability of the birnessite nanosheets.

Although currently the most popular current collector and mechanical support for electrodes in supercapacitors is aluminum foil, sometimes treated mechanically or chemically for slightly modified surface properties to improve the adhesion of the active mass and reduce electrical contact resistance (not electrochemical charge transfer resistance as sometimes erroneously claimed), other materials have been examined. Better mechanical properties and higher corrosion stability are among the arguments for proposing thin foils or meshes of stainless steel of various compositions. Coating with high-quality graphene by direct deposition in a CVD process has been studied [166]. Improved electric contact, i.e., lower Ohmic resistance, was observed as the desired effect of this surface modification, which was claimed as being suitable also for coating other current collector materials. As a consequence, all of the related performance properties of an EDLC supercapacitor were noticeably improved.

A coating with graphene or graphene oxide—the report remains ambiguous about this difference—on polyacrylonitrile fiber cloth was applied before carbonization [167]. The amount of initially deposited graphene oxide had some influence on the relative performance improvements of the prepared electrodes; in the absence of a blank (without such coating), the observed effects are hard to attribute to the coating in detail. The authors claimed a reduced specific surface area because of the “shielding effect” of the coating presumably during carbonization and subsequent chemical activation.

The capacitive behavior of carbon nanofiber cloth coated with GO was examined [168]. The somewhat ambiguous description does not reveal the intended function; the absence of data obtained without such a coating does not help.

Passivating the graphene coating of porous silicon to be used as an electrode material in supercapacitors has been reviewed, and higher electronic conductivity essential for this application was observed [159].

Graphene coatings of textiles were used to provide surface conductivity for subsequent deposition of MnO_2_ in an otherwise barely comprehensible report on flexible supercapacitors [169]. Coatings of rGO were applied in supercapacitor-related studies reported in an otherwise mysterious report [170].

Various coatings of carbon cloth with carbon-coated aluminum foil, used as a support and current collector in supercapacitors, were compared, and coating with graphene yielded superior results in terms of recorded capacitance and internal cell resistance [171].

### 2.3. Coatings in Fuel Cells

The layered Ni-Co double hydroxides with an ultrathin conformal graphene coating already mentioned above as a negative electrode material for lithium-ion batteries [78] also provided significantly enhanced electrocatalytic activity in the oxygen evolution reaction.

Thermally annealed self-assembled three-dimensional graphene was proposed as a cheaper substitute of CVD-coated graphene on porous metal foam in PEM fuel cells, yielding improved cell performance [172].

Graphene coatings on metals, alloys, and other materials for bipolar plates for PEM fuel cells showed high corrosion protection [173], and their further advantages were reviewed [174,175]. The catalytic properties of the metal components of the alloys were effective through the very thin graphene coating. The degradation mechanisms of graphene coatings on bipolar plates for PEM fuel cells were reviewed [176]. Fabrication defects, acting as initiation sites for degradation, were highlighted. Degradation of graphene-coated copper in a simulated PEMFC environment was studied using spectroscopic methods [177]. Coated copper had significantly higher corrosion resistance after extended exposure to the simulated environment, confirming the corrosion-protective effect.

The catalytic effects of graphene coatings on various crystallographic platinum surfaces in the dioxygen reduction reaction were studied via DFT calculations [178]. Further enhancement of catalytic activity by nitrogen doping appeared to be feasible. Carbon nanotube and graphene coatings on stainless steel mesh, used as the positive electrode (dioxygen reduction electrode) in a microbial fuel cell, increased the power density and decreased the internal resistance of the cell [179].

Graphene-coated nickel foam, used as the negative electrode (the authors use the term cathode) in microbial electrosynthesis of acetate from CO_2_, yielded a 1.8-fold increase in volumetric acetate production as compared to the uncoated nickel foam [180]. Both increased active surface area (see also [181,182,183]) and accelerated electron transfer were invoked as reasons.

### 2.4. Further Applications

With redox flow batteries (for an introduction, see [12]), graphene coating of the Nafion^®^ membrane to separate the two electrolyte solutions in the half cells was reported [184]. In a single-cell study, higher energy efficiency, power density, and discharge capacity were recorded. These benefits were attributed to reduced vanadium crossover and enhanced electrochemical activity. Both arguments remain mysterious in the light of a recently reported major survey [185]. Similar benefits were reported for devices employed in flow-electrode capacitive mixing [186].

## 3. Conclusions

Graphene and its chemical relatives, graphene oxide, reduced graphene oxide, and few-layer graphene (flG), coated by different methods on active masses and auxiliary components in systems for EET are suggested and examined. The beneficial effects and their conceivable reasons and modes of operation are reviewed. At very low actual contents of the coating material, the beneficial effects are established mostly by increased capacitance, higher current capability, and improved stability. The suggested reasons for the improvement of electrode and material performance and mode of operation are as follows:

- Protection of the active mass against dissolution and/or corrosion;
- Increased electronic conductance;
- Mitigation of volume change effects;
- Inhibition of pulverization and agglomeration.

Further research should aim at the optimization of coating procedures and the amounts of added material based on a deeper understanding of the mode of operation. Longer stability tests aiming at higher cycle numbers closer to commercial expectations are urgently needed.

## Figures and Tables

**Table 1:** Literature values of electronic conductivity of graphene and related materials.

Material	Electronic Conductivity	References
graphite	3.14·10^3^ S/cm ^1^	[4]
graphite	2.0·10^3^ to 4.0·10^3^ S/cm	[5]
graphene	6000 S/cm to 100 MS/m	various
graphene	1.46 ± 0.82·10^6^ S/m.	[6]
single-layer graphene	7.14·10^5^ S/cm	[4]
single-layer graphene	1.0·10^6^ S/cm	[7,8]
few-layer graphene	1.22·10^5^ to 5.26·10^5^ S/cm ^1,2^	[4]
few-layer graphene	2.94·10^5^ to 8.33·10^5^ S/cm	[9,10]
graphene nanosheets	1.0^5^ to 6.0^3^ S/cm ^1,2^	[4]
graphene oxide	4.57 × 10^−8^ S/cm	[11]
reduced graphene oxide	2.3 ± 1.0 to 14.6 ± 5.5 S/m	[5]
reduced graphene oxide	4.21 × 10^−5^ S/cm	[6]

^1^ Calculated values; ^2^ Value depends on number of layers.

## References

[1] Zhao X., E J., Wu G., Deng Y., Han D., Zhang B., Zhang Z. (2019). A review of studies using graphenes in energy conversion, energy storage and heat transfer development. Energy Conv. Managem..

[2] Xu H., Zeng M., Li J. (2015). Graphene-wrapped Cr_2_O_3_ hollow nanospheres with enhanced electrochemical performances for lithium-ion batteries. Int. J. Electrochem. Sci..

[3] Ul Hoque M.I., Donne S.W., Holze R. (2024). Graphene Nanocomposite Materials for Supercapacitor Electrodes. Encyclopedia.

[4] Fang X.Y., Yu X.X., Zheng H.M., Jin H.B., Wang L., Cao M.S. (2015). Temperature- and thickness-dependent electrical conductivity of few-layer graphene and graphene nanosheets. Phys. Lett. A.

[5] Hugh O.P. (1993). Handbook of Carbon, Graphite, Diamond, and Fullerenes: Properties, Processing, and Applications.

[6] Lim S., Park H., Yamamoto G., Lee C., Suk J.W. (2021). Measurements of the electrical conductivity of monolayer graphene flakes using conductive atomic force microscopy. Nanomaterials.

[7] Geim A.K., Novoselov K.S. (2007). The rise of graphene. Nat. Mater..

[8] Berger C., Song Z., Li X., Wu X., Brown N., Naud C., Mayou D., Li T., Haas J., Marchenkov A. (2006). Electronic confinement and coherence in patterned epitaxial graphene. Science.

[9] Rouhi N., Wang Y.Y., Burke P.J. (2012). Electrical Conductivity of Graphene Composites with In and In-Ga Alloy. Appl. Phys. Lett..

[10] Sruti A.N., Jagannadham K. (2010). Ultrahigh conductivity of large area suspended few layer graphene films. J. Electron. Mater..

[11] Jaafar E., Kashif M., Sahari S.K., Ngaini Z. (2018). Study on morphological, optical and electrical properties of graphene oxide (GO) and reduced graphene oxide (rGO). Mater. Sci. Forum.

[12] Wu Y., Holze R. (2022). Electrochemical Energy Conversion and Storage.

[13] Xie X., Holze R. (2023). Corrosion in supercapacitors—An Overview. Univ. J. Electrochem..

[14] Chen X., Wu Y., Holze R. (2023). Ag(e)ing and Degradation of Supercapacitors: Causes, Mechanisms, Models and Countermeasures. Molecules.

[15] Liu X.H., Liu Y., Kushima A., Zhang S., Zhu T., Li J., Huang J.H. (2012). In situ TEM experiments of electrochemical lithiation and delithiation of individual nanostructures. Adv. Energy Mater..

[16] Mi H., Li F., Xu S., Li Z., Chai X., He C., Li Y., Liu J. (2016). A Tremella-Like Nanostructure of Silicon@void@graphene-Like Nanosheets Composite as an Anode for Lithium-Ion Batteries. Nanoscale Res. Lett..

[17] Zhang J.P., Wu X.Y., Wei X., Xu S.M., Ma C., Shu M.H., Wang K.X., Chen J.S. (2018). Top-down fabrication of hierarchical nanocubes on nanosheets composite for high-rate lithium storage. Dalton Trans..

[18] Hu G., Wu J., Du K., Peng Z., Jia M., Yang H., Cao Y. (2019). Surface-fluorinated Li_4_Ti_5_O_12_ nanowires/reduced graphene oxide composite as a high-rate anode material for Lithium ion batteries. Appl. Surf. Sci..

[19] Zhang L., Ge D., Geng H., Zheng J., Cao X., Gu H. (2017). Synthesis of porous Mn_2_O_3_ embedded in reduced graphene oxide as advanced anode materials for lithium storage. New J. Chem..

[20] Baboo J.P., Babar S., Kale D., Lekakou C., Laudone G.M. (2021). Designing a graphene coating-based supercapacitor with lithium ion electrolyte: An experimental and computational study via multiscale modeling. Nanomaterials.

[21] Zhou F., Zeng L., Guo W., Yan Y., Wu F., Pan M. (2019). Enhanced performance of alpha-Fe_2_O_3_ nanoparticles with optimized graphene coated layer as anodes for lithium-ion batteries. Int. J. Energy Res..

[22] Leng W., Cui L., Liu Y., Gong Y. (2022). MOF-Derived MnV_2_O_4_/C Microparticles with Graphene Coating Anchored on Graphite Sheets: Oxygen Defect Engaged High Performance Aqueous Zinc-Ion Battery. Adv. Mater. Interfaces.

[23] Jeon J.W., Kwon S.R., Lutkenhaus J.L. (2015). Polyaniline nanofiber/electrochemically reduced graphene oxide layer-by-layer electrodes for electrochemical energy storage. J. Mater. Chem. A.

[24] Zhao C., Luo X., Chen C., Wu H. (2016). Sandwich electrode designed for high performance lithium-ion battery. Nanoscale.

[25] Liu D., Xie X., Chen X., Holze R. (2025). Graphene for corrosion protection in electrochemical energy technology. Corros. Mater. Degrad..

[26] Ranjan H., Ranjan P., Sahu T.K., Sharma R.K., Kumar P. (2023). Reduced graphene oxide electrode-coating as anti-corrosive/anti-oxidative laminate for Al/Cu liquid-phase batteries. J. Mater. Res..

[27] Fu L., Qu Q., Holze R., Kondratiev V.V., Wu Y. (2019). Composites of metal oxides and intrinsically conducting polymers as supercapacitor electrode materials: The best of both worlds?. J. Mater. Chem. A.

[28] Chen J., Bie L., Sun J., Xu F. (2016). Enhanced electrochemical performances of silicon nanotube bundles anode coated with graphene layers. Mater. Res. Bull..

[29] Xu S., Zhou J., Zuin L., Sun D., Zhao J., Bellal A., Hou X. (2024). Synthesis of hierarchical graphene coated porous Si anode for lithium-ion batteries. J. Energy Storage.

[30] Li L., Yang Y., Huang Z., Huang T., Chen W., Gong X., Ye S., Li H., Huang S., Xiong W. (2024). Hydrogen bond interaction derived homogeneous graphene coating on submicron silicon anode. Battery Energy.

[31] Kim S.C., Huang E., Zhang Z., Wang J., Kim Y., Jeong Y.K., Oyakhire S.T., Yang Y., Cui Y. (2022). Graphene coating on silicon anodes enabled by thermal surface modification for high-energy lithium-ion batteries. MRS Bull..

[32] Son I.H., Park J.H., Kwon S., Park S., Rümmeli M.H., Bachmatiuk A., Song H.J., Ku J., Choi J.W., Choi J.M. (2015). Silicon carbide-free graphene growth on silicon for lithium-ion battery with high volumetric energy density. Nat. Commun..

[33] Son I.H., Park J.H., Kwon S., Choi J.W., Rümmeli M.H. (2016). Graphene coating of silicon nanoparticles with CO_2_-enhanced chemical vapor deposition. Small.

[34] Wang G., Xu B., Shi J., Lei X., Ouyang C. (2018). Confined Li ion migration in the silicon-graphene complex system: An ab initio investigation. Appl. Surf. Sci..

[35] Wang C., Luo F., Lu H., Liu B., Chu G., Quan B., Li J., Gu C., Li H., Chen L. (2017). Side-by-side observation of the interfacial improvement of vertical graphene-coated silicon nanocone anodes for lithium-ion batteries by patterning technology. Nanoscale.

[36] Chang C., Li X., Xu Z., Gao H. (2017). Lithiation-enhanced charge transfer and sliding strength at the silicon-graphene interface: A first-principles study. Acta Mechan. Sol. Sin..

[37] Liu Z., Huang J., Zhao X., Huang H., Fu C., Li Z., Cheng Y., Niu C., Zhang J. (2019). A Facile Path to Graphene-Wrapped Polydopamine-Entwined Silicon Nanoparticles with High Electrochemical Performance. ChemPlusChem.

[38] Khan A., Kumar R.R., Cong J., Imran M., Yang D., Yu X. (2022). CVD Graphene on Textured Silicon: An Emerging Technologically Versatile Heterostructure for Energy and Detection Applications. Adv. Mater. Interfaces.

[39] Wasalathilake K.C., Hapuarachchi S.N.S., Zhao Y., Fernando J.F.S., Chen H., Nerkar J.Y., Golberg D., Zhang S., Yan C. (2020). Unveiling the Working Mechanism of Graphene Bubble Film/Silicon Composite Anodes in Li-Ion Batteries: From Experiment to Modeling. ACS Appl. Energy Mater..

[40] Park J.H., Moon J., Han S., Park S., Lim J.W., Yun D.J., Kim D.Y., Park K., Son I.H. (2017). Formation of Stable Solid-Electrolyte Interphase Layer on Few-Layer Graphene-Coated Silicon Nanoparticles for High-Capacity Li-Ion Battery Anodes. J. Phys. Chem. C.

[41] Luo J., Zhao X., Wu J., Jang H.D., Kung H.H., Huang J. (2012). Crumpled graphene-encapsulated Si nanoparticles for lithium ion battery anodes. J. Phys. Chem. Lett..

[42] Lin N., Zhou J., Wang L., Zhu Y., Qian Y. (2015). Polyaniline-assisted synthesis of Si@C/RGO as anode material for rechargeable lithium-ion batteries. ACS Appl. Mater. Interfaces.

[43] Jin Y., Tan Y., Hu X., Zhu B., Zheng Q., Zhang Z., Zhu G., Yu Q., Jin Z., Zhu J. (2017). Scalable Production of the Silicon-Tin Yin-Yang Hybrid Structure with Graphene Coating for High Performance Lithium-Ion Battery Anodes. ACS Appl. Mater. Interfaces.

[44] Xu S., Zhou J., Wang J., Pathiranage S., Oncel N., Robert Ilango P., Zhang X., Mann M., Hou X. (2021). In Situ Synthesis of Graphene-Coated Silicon Monoxide Anodes from Coal-Derived Humic Acid for High-Performance Lithium-Ion Batteries. Adv. Funct. Mater..

[45] Li Z., Tao X., Yang Y., Yao N., Yang Z., Luo D., Wang J., Zhao H. (2022). Enhanced cycling performance of SiOx microparticles uniformly coated with graphene sheets. Electrochim. Acta.

[46] Dang G., Zhang M., Min F., Zhang Q., Lv T., Liu W., Wang J., Zhou Y., Xie J., Mao S.S. (2025). Unleashing the potential: SiOx@GNs composites for superior lithium-ion battery anodes. J. Materiom..

[47] Gu H., Wang Y., Zeng Y., Yu M., Liu T., Chen J., Wang K., Xie J., Li L. (2022). Boosting Cyclability and Rate Capability of SiOx via Dopamine Polymerization-Assisted Hybrid Graphene Coating for Advanced Lithium-Ion Batteries. ACS Appl. Mater. Interfaces.

[48] Jin S., Li N., Cui H., Wang C. (2014). Embedded into graphene Ge nanoparticles highly dispersed on vertically aligned graphene with excellent electrochemical performance for lithium storage. ACS Appl. Mater. Interfaces.

[49] Zhou X., Yin Y.X., Wan L.J., Guo Y.G. (2012). A robust composite of SnO_2_ hollow nanospheres enwrapped by graphene as a high-capacity anode material for lithium-ion batteries. J. Mater. Chem..

[50] Zhou D., Li X., Fan L.Z., Deng Y. (2017). Three-dimensional porous graphene-encapsulated CNT@SnO_2_ composite for high-performance lithium and sodium storage. Electrochim. Acta.

[51] Deng L., Zhu J., Chen X., Ding M., Liu H. (2018). Three-dimensional elastic ultrathin reduced graphene oxide coating SnS2 hierarchical microsphere as lithium ion batteries anode materials. J. Alloys Compd..

[52] Liu H.P., Wen G.W., Bi S.F., Wang C.Y., Hao J.M., Gao P. (2016). High rate cycling performance of nanosized Li_4_Ti_5_O_12_/graphene composites for lithium ion batteries. Electrochim. Acta.

[53] Ding Z., Zhao L., Suo L., Jiao Y., Meng S., Hu Y.S., Wang Z., Chen L. (2011). Towards understanding the effects of carbon and nitrogen-doped carbon coating on the electrochemical performance of Li_4_Ti_5_O_12_ in lithium ion batteries: A combined experimental and theoretical study. Phys. Chem. Chem. Phys..

[54] Wu H., Gan Y., Yao Q., Wang L.P., Wang C., Zhang Q., Hou K., Zhao Y., Guan L. (2020). Boosting the lithium and sodium storage performance of graphene-based composite via pore engineering and surface protection. Nanotechnology.

[55] Tong X., Zeng M., Xu H., Li J. (2016). Synthesis and lithium storage performance of graphene/Co_3_O_4_ microrods hybrids. J. Mater. Sci.: Mater. Electron..

[56] Wu K., Geng B., Zhang C., Shen W., Yang D., Li Z., Yang Z., Pan D. (2020). Hierarchical porous arrays of mesoporous Co_3_O_4_ nanosheets grown on graphene skin for high-rate and high-capacity energy storage. J. Alloys Compd..

[57] Sadeghi Ghazvini A.A., Taheri-Nassaj E., Raissi B., Riahifar R., Sahba Yaghmaee M., Shaker M. (2021). Co-electrophoretic deposition of Co3O4 and graphene nanoplates for supercapacitor electrode. Mater. Lett..

[58] Kang C., Cha E., Lee S.H., Choi W. (2018). In situ fabrication of a graphene-coated three-dimensional nickel oxide anode for high-capacity lithium-ion batteries. RSC Adv..

[59] Park K.Y., Zhu Y., Torres-Castanedo C.G., Jung H.J., Luu N.S., Kahvecioglu O., Yoo Y., Seo J.W.T., Downing J.R., Lim H.D. (2022). Elucidating and Mitigating High-Voltage Degradation Cascades in Cobalt-Free LiNiO_2_ Lithium-Ion Battery Cathodes. Adv. Mater..

[60] Chen K.S., Xu R., Luu N.S., Secor E.B., Hamamoto K., Li Q., Kim S., Sangwan V.K., Balla I., Guiney L.M. (2017). Comprehensive Enhancement of Nanostructured Lithium-Ion Battery Cathode Materials via Conformal Graphene Dispersion. Nano Lett..

[61] Sharifi-Asl S., Soto F.A., Foroozan T., Asadi M., Yuan Y., Deivanayagam R., Rojaee R., Song B., Bi X., Amine K. (2019). Anti-Oxygen Leaking LiCoO_2_. Adv. Funct. Mater..

[62] Zhang Y., Zheng S., Meng C., Liu H., Dong C., Shi X., Das P., Huang R., Yu Y., Wu Z.S. (2023). A Near-Surface Structure Reconfiguration Strategy to Regulate Mn^3+^/Mn^4+^ and O^2-^/(O_2_)^n-^ Redox for Stabilizing Lithium-Rich Oxide Cathode. Adv. Funct. Mater..

[63] Dai H., Zhang R., Zhong M., Guo S. (2020). Effects of the Inherent Tubular Structure and Graphene Coating on the Lithium Ion Storage Performances of Electrospun NiO/Co_3_O_4_ Nanotubes. J. Phys. Chem. C.

[64] Tao J., Liu G., Chen Y., Chi Y., Hong L., Lin Z., Lin Y., Huang Z. (2018). 3D plum candy-like NiCoMnO4@graphene as anodes for high-performance lithium-ion batteries. RSC Adv..

[65] Chen H., He P., Li M., Wen Y., Cao G., Qiu J., Ming H., Zhao P., Zhang S. (2021). Bifunctional Sulfonated Graphene-Modified LiNi_0.5_Mn_1.5_O_4_ for Long-Life and High-Energy-Density Lithium-Ion Batteries. ACS Appl. Energy Mater..

[66] Gong F., Xia D., Bi C., Yang J., Zeng W., Chen C., Ding Y., Xu Z., Liao J., Wu M. (2018). Systematic comparison of hollow and solid Co_3_V_2_O_8_ micropencils as advanced anode materials for lithium ion batteries. Electrochim. Acta.

[67] Gao M., Liu X., Yang Y., Yu Y. (2018). FeP nanoparticles derived from metal-organic frameworks/GO as high-performance anode material for lithium ion batteries. Sci. China Chem..

[68] Jing P., Wang Q., Xian C., Du L., Zhang Y., Wang B., Wu H., Wu K., Wang Q., Zhang Y. (2021). Ultrafast and durable Li/Na storage by an iron selenide anode using an elastic hierarchical structure. Inorg. Chem. Front..

[69] Zhang B., Xu Z.L., Kim J.K. (2014). In situ grown graphitic carbon/Fe_2_O_3_/carbon nanofiber composites for high performance freestanding anodes in Li-ion batteries. RSC Adv..

[70] Li X., Zheng X., Shao J., Gao T., Shi Q., Qu Q. (2016). Synergistic Ternary Composite (Carbon/Fe_3_O_4_@Graphene) with Hollow Microspherical and Robust Structure for Li-Ion Storage. Chem. Eur. J..

[71] Xu W., Xue W., Zhang Y., Zhang B., Wang Y., Zhao R. (2018). Graphene coating magnetite/N-doping carbon hybrid composites and its lithium storage performance. Mater. Lett..

[72] Zhang M., Zhao J., Fang Z., Wu M. (2023). Alkali ions pre-intercalation and reduced graphene coating of MnO_2_ for high-capacity Li-ion battery. E3S Web Conf..

[73] Lyu D., Zhang L., Wei H., Geng H., Gu H. (2017). Synthesis of graphene wrapped porous CoMoO_4_ nanospheres as high-performance anodes for rechargeable lithium-ion batteries. RSC Adv..

[74] Chen K., Zhang F., Sun J., Li Z., Zhang L., Bachmatiuk A., Zou Z., Chen Z., Zhang L., Rümmeli M.H. (2018). Growth of defect-engineered graphene on manganese oxides for Li-ion storage. Energy Stor. Mater..

[75] Wang T., Kong Z., Guo F., Liu X., Fu A., Li Y., Guo P., Guo Y.G., Li H. (2020). Graphene-encapsulated ZnO composites as high-performance anode materials for lithium ion batteries. Ionics.

[76] Zhang J., Zhao Y., Zhang Y., Li J., Babaa M.R., Liu N., Bakenov Z. (2020). Synthesis of microflower-like vacancy defective copper sulfide/reduced graphene oxide composites for highly efficient lithium-ion batteries. Nanotechnology.

[77] Fedoseeva Y.V., Makarova A.A., Stolyarova S.G., Arkhipov V.E., Rühl E., Okotrub A.V., Bulusheva L.G. (2022). Lithium-induced intralayer rearrangement of molybdenum disulfide: Effect of graphene coating. Appl. Surf. Sci..

[78] Shi J., Du N., Zheng W., Li X., Dai Y., He G. (2017). Ultrathin Ni-Co double hydroxide nanosheets with conformal graphene coating for highly active oxygen evolution reaction and lithium ion battery anode materials. Chem. Eng. J..

[79] Xu T., Sun W., Kong T., Zhou J., Qian Y. (2024). Stable Graphite Interface for Potassium Ion Battery Achieving Ultralong Cycling Performance. Acta Phys. Chim. Sin..

[80] Ma Y., Qi P., Ma J., Wei L., Zhao L., Cheng J., Su Y., Gu Y., Lian Y., Peng Y. (2021). Wax-Transferred Hydrophobic CVD Graphene Enables Water-Resistant and Dendrite-Free Lithium Anode toward Long Cycle Li-Air Battery. Adv. Sci..

[81] Yu Y., Ying D., Xu S., Guo Q., Li Y., Wang S., Zhou X., Shao G., Liu Z. (2023). Graphene coated lithium foil anode enables long cycle life Li metal pouch cells. Carbon.

[82] Bai M., Xie K., Yuan K., Zhang K., Li N., Shen C., Lai Y., Vajtai R., Ajayan P., Wei B. (2018). A Scalable Approach to Dendrite-Free Lithium Anodes via Spontaneous Reduction of Spray-Coated Graphene Oxide Layers. Adv. Mater..

[83] Zhang R., Wen S., Wang N., Qin K., Liu E., Shi C., Zhao N. (2018). N-Doped Graphene Modified 3D Porous Cu Current Collector toward Microscale Homogeneous Li Deposition for Li Metal Anodes. Adv. Energy Mater..

[84] Kim J.S., Kim D.W., Jung H.T., Choi J.W. (2015). Controlled Lithium Dendrite Growth by a Synergistic Effect of Multilayered Graphene Coating and an Electrolyte Additive. Chem. Mater..

[85] Ren F., Peng Z., Wang M., Xie Y., Li Z., Wan H., Lin H., Wang D. (2019). Over-potential induced Li/Na filtrated depositions using stacked graphene coating on copper scaffold. Energy Stor. Mater..

[86] Tang S., Luo D., Bai S., Wu M., Zhang J., Liu W., Yang Z. (2022). Unravelling the regulating role of graphene coating on improving the electrochemical performance of pyrophosphate cathode material: A first-principles study. Appl. Surf. Sci..

[87] Tian Z., Liu S., Ye F., Yao S., Zhou Z., Wang S. (2014). Synthesis and characterization of LiFePO_4_ electrode materials coated by graphene. Appl. Surf. Sci..

[88] Li J., Zhang L., Zhang L., Hao W., Wang H., Qu Q., Zheng H. (2014). In-situ growth of graphene decorations for high-performance LiFePO_4_ cathode through solid-state reaction. J. Power Sources.

[89] Hang L.T., Anh N.T.M., Tru N.N., Nguyen H.L.T., Phung L.M.L. (2018). Modification of nanosized LiFePO_4_ via nickel doping and graphene coating. Int. J. Nanotechnol..

[90] Tian Z., Zhou Z., Liu S., Ye F., Yao S. (2015). Enhanced properties of olivine LiFePO_4_/graphene co-doped with Nb^5+^ and Ti^4+^ by a sol-gel method. Solid State Ionics.

[91] Zhang C., Lou J., Li J., Song J., Qi Z., Huo S., Lin Y., Yang F., Liu L. (2024). Graphene coating-modified LiCoO_2_ films as high-performance cathode material in quasi-solid-state thin-film lithium batteries. Appl. Surf. Sci..

[92] Holze R. (2024). Between the Electrodes of a Supercapacitor: An Update on Electrolytes. Adv. Mater. Sci. Technol..

[93] Chen X., Holze R. (2024). Polymer Electrolytes for Supercapacitors. Polymers.

[94] Ding D., Maeyoshi Y., Kubota M., Wakasugi J., Kanamura K., Abe H. (2019). Highly improved performances of LiMn_0.7_Fe_0.3_PO_4_ cathode with in situ electrochemically reduced graphene oxide. J. Alloys Compd..

[95] Liu L., Zhang H., Chen X., Fang L., Bai Y., Liu R., Wang Y. (2015). Unique synthesis of sandwiched graphene@(Li_0.893_Fe_0.036_)Co(PO_4_) nanoparticles as high-performance cathode materials for lithium-ion batteries. J. Mater. Chem. A.

[96] Yang J., Kang X., He D., Zheng A., Pan M., Mu S. (2015). Graphene activated 3D-hierarchical flower-like Li_2_FeSiO_4_ for high-performance lithium-ion batteries. J. Mater. Chem. A.

[97] Loftager S., García-Lastra J.M., Vegge T. (2017). A density functional theory study of the carbon-coating effects on lithium iron borate battery electrodes. Phys. Chem. Chem. Phys..

[98] Jaber-Ansari L., Puntambekar K.P., Kim S., Aykol M., Luo L., Wu J., Myers B.D., Iddir H., Russell J.T., Saldaña S.J. (2015). Suppressing Manganese Dissolution from Lithium Manganese Oxide Spinel Cathodes with Single-Layer Graphene. Adv. Energy Mater..

[99] Gao C., Liu H., Bi S., Fan S., Meng X., Li Q., Luo C. (2020). Insight into the effect of graphene coating on cycling stability of LiNi_0.5_Mn_1.5_O_4_: Integration of structure-stability and surface-stability. J. Materiom..

[100] Gao C., Liu H., Bi S., Fan S., Liu Q., Li H., Cao L., Luo C. (2020). Insight into the High-Temperature Cycling Stability of a Micro-nanostructured LiNi_0.5_Mn_1.5_O_4_/Graphene Composite Cathode for High-Voltage Lithium-Ion Batteries. J. Phys. Chem. C.

[101] Xu H., Ai L., Yan J., Yan G., Zhang W. (2019). Enhanced electrochemical performance of LiNi_0.5_Co_0.2_Mn_0.3_O_2_ cathodes by cerium doping and graphene coating. Ceram. Int..

[102] Wu F., Yan Y., Wang R., Cai H., Tong W., Tang H. (2017). Synthesis of LiNi_1/3_Mn_1/3_Co_1/3_O_2_@graphene for lithium-ion batteries via self-assembled polyelectrolyte layers. Ceram. Int..

[103] Ning R., Yuan K., Zhang K., Shen C., Xie K. (2021). A scalable snowballing strategy to construct uniform rGO-wrapped LiNi_0.8_Co_0.1_Mn_0.1_O_2_ with enhanced processability and electrochemical performance. Appl. Surf. Sci..

[104] Hwang J., Lee S., Kim S., Do K., Kim S., Jo H., Lim H.D., Ahn H. (2023). Uniform and Multifunctional PEI-POSS/Carbon Encapsulation for High-Rate Performance and Surface Stabilization of Nickel-Rich Layered Cathodes in Lithium-Ion Batteries. Adv. Funct. Mater..

[105] Ge Y., Liu Z., Wu Y., Holze R. (2021). On the utilization of supercapacitor electrode materials. Electrochim. Acta.

[106] Park K.Y., Lim J.M., Luu N.S., Downing J.R., Wallace S.G., Chaney L.E., Yoo H., Hyun W.J., Kim H.U., Hersam M.C. (2020). Concurrently Approaching Volumetric and Specific Capacity Limits of Lithium Battery Cathodes via Conformal Pickering Emulsion Graphene Coatings. Adv. Energy Mater..

[107] Xue B., Wu X. (2023). High structural stability of graphene coated nickel—Rich cathode material in Li—Ion battery. J. Alloys Compd..

[108] Zhang J., He H., Wang X., Mao G., Yu W., Ding Z., Tian Q., Tong H., Guo X. (2022). A new modification strategy for improving the electrochemical performance of high-nickel cathode material: V_2_O_5_ particles anchored on rGO sheets as a dual coating layer. Appl. Surf. Sci..

[109] Lim J.M., Luu N.S., Park K.Y., Tan M.T.Z., Kim S., Downing J.R., He K., Dravid V.P., Hersam M.C. (2020). Enhancing nanostructured nickel-rich lithium-ion battery cathodes via surface stabilization. J. Vac. Sci. Technol. A.

[110] Luu N.S., Lim J.M., Torres-Castanedo C.G., Park K.Y., Moazzen E., He K., Meza P.E., Li W., Downing J.R., Hu X. (2021). Elucidating and Mitigating High-Voltage Interfacial Chemomechanical Degradation of Nickel-Rich Lithium-Ion Battery Cathodes via Conformal Graphene Coating. ACS Appl. Energy Mater..

[111] Ning F., Shang H., Li B., Jiang N., Zou R., Xia D. (2019). Surface thermodynamic stability of Li-rich Li_2_MnO_3_: Effect of defective graphene. Energy Stor. Mater..

[112] Kim T., Song B., Lunt A.J.G., Cibin G., Dent A.J., Lu L., Korsunsky A.M. (2016). In operando X-ray absorption spectroscopy study of charge rate effects on the atomic environment in graphene-coated Li-rich mixed oxide cathode. Mater. Design.

[113] Kim T., Song B., Cibin G., Dent A., Li L., Korsunsky A.M. (2014). A comparative spectroscopic study of graphene-coated vs pristine Li(Mn, Ni, Co) oxide materials for lithium-ion battery cathodes. Proc. Int. MultiConf. Eng. Comp. Sci..

[114] He Z., Wang Z., Guo H., Li X., Xianwen W., Yue P., Wang J. (2013). A simple method of preparing graphene-coated Li[Li_0.2_Mn _0.54_Ni_0.13_Co_0.13_]O_2_ for lithium-ion batteries. Mater. Lett..

[115] Zhu J., Gandi A.N., Gu M. (2018). Phosphorene as cathode for metal-ion batteries: Importance of F decoration. Mater. Today Energy.

[116] Liu Y., Guo J., Zhang J., Su Q., Du G. (2015). Graphene-wrapped sulfur nanospheres with ultra-high sulfur loading for high energy density lithium-sulfur batteries. Appl. Surf. Sci..

[117] Wang H., Yang H., Liang Y., Robinson J.T., Li Y., Jackson A., Cui Y., Dai H. (2011). Graphene-wrapped sulfur particles as a rechargeable lithium-sulfur battery cathode material with high capacity and cycling stability. Nano Lett..

[118] Wei M., Yuan P., Chen W., Hu J., Mao J., Shao G. (2015). Facile assembly of partly graphene-enveloped sulfur composites in double-solvent for lithium-sulfur batteries. Electrochim. Acta.

[119] Chen S., Tang Q., Chen X., Hu A., Deng W., Liu Z. (2015). Controllable graphene coated mesoporous carbon/sulfur composite for lithium-sulfur batteries. RSC Adv..

[120] Zhou X., Xie J., Yang J., Zou Y., Tang J., Wang S., Ma L., Liao Q. (2013). Improving the performance of lithium-sulfur batteries by graphene coating. J. Power Sources.

[121] Zhao X.Y., Tu J.P., Lu Y., Cai J.B., Zhang Y.J., Wang X.L., Gu C.D. (2013). Graphene-coated mesoporous carbon/sulfur cathode with enhanced cycling stability. Electrochim. Acta.

[122] Zheng J.F., Zheng M.B., Li N.W., Lu H.L., Qiu L., Cao J.M., Ji G.B. (2013). Preparation of graphene coated carbon nanotube-sulfur composite and its performance for lithium-sulfur battery. Chin. J. Inorg. Chem..

[123] Xie J., Yang J., Zhou X., Zou Y., Tang J., Wang S., Chen F. (2014). Preparation of three-dimensional hybrid nanostructure-encapsulated sulfur cathode for high-rate lithium sulfur batteries. J. Power Sources.

[124] Guo Y., Wu H., Zhang Y., Xiang M., Zhao G., Liu H., Zhang Y. (2017). Vesicle-like sulfur/reduced graphene oxide composites for high performance lithium-sulfur batteries. J. Alloys Compd..

[125] Tang X.N., Sun Z.H., Zhuo S.P., Li F. (2017). Nitrogen-doped CMK-3@graphene hybrids as a sulfur host material for use in lithium-sulfur batteries. New Carb. Mater..

[126] Zhou X., Chen F., Yang J., Ma L., Bai T., Long B., Liao Q., Liu C. (2015). Dual protection of sulfur by interconnected porous carbon nanorods and graphene sheets for lithium-sulfur batteries. J. Electroanal. Chem..

[127] Zhou X., Chen F., Yang J. (2015). Core@shell sulfur@polypyrrole nanoparticles sandwiched in graphene sheets as cathode for lithium-sulfur batteries. J. Energy Chem..

[128] Cengiz E., Salihoglu O., Ozturk O., Kocabas C., Demir-Cakan R. (2019). Ultra-lightweight Chemical Vapor Deposition grown multilayered graphene coatings on paper separator as interlayer in lithium-sulfur batteries. J. Alloys Compd..

[129] Guo J., Jiang H., Li X., Chu Z., Zheng W., Dai Y., Jiang X., Wu X., He G. (2021). Defective graphene coating-induced exposed interfaces on CoS nanosheets for high redox electrocatalysis in lithium-sulfur batteries. Energy Stor. Mater..

[130] Yang J., Cao C., Qiao W., Qiao J., Tang C., Xue Y. (2023). B/N co-doping rGO/BNNSs heterostructure with synergistic adsorption-electrocatalysis function enabling enhanced electrochemical performance of lithium-sulfur batteries. Chem. Eng. J..

[131] Wang L., He Y.B., Shen L., Lei D., Ma J., Ye H., Shi K., Li B., Kang F. (2018). Ultra-small self-discharge and stable lithium-sulfur batteries achieved by synergetic effects of multicomponent sandwich-type composite interlayer. Nano Energy.

[132] Kim S.Y., Song Y.I., Wee J.H., Kim C.H., Ahn B.W., Lee J.W., Shu S.J., Terrones M., Kim Y.A., Yang C.M. (2019). Few-layer graphene coated current collector for safe and powerful lithium ion battery. Carbon.

[133] Zhou Y., Wang H., Wang J. (2024). Composite Graphene-Modified Aluminum Foil Cathode Current Collectors for Lithium-ion Battery with Enhanced Mechanical and Electrochemical Performances. Batteries&Supercaps.

[134] Wang R., Li W., Liu L., Qian Y., Liu F., Chen M., Guo Y., Liu L. (2019). Carbon black/graphene-modified aluminum foil cathode current collectors for lithium ion batteries with enhanced electrochemical performances. J. Electroanal. Chem..

[135] Jiang J., Nie P., Ding B., Wu W., Chang Z., Wu Y., Dou H., Zhang X. (2016). Effect of Graphene Modified Cu Current Collector on the Performance of Li4Ti5O12 Anode for Lithium-Ion Batteries. ACS Appl. Mater. Interfaces.

[136] Liu G., Xie J., Sun Y., Zhang P., Li X., Zheng L., Hao L., Shanmin G. (2021). Constructing 3D honeycomb-like CoMn_2_O_4_ nanoarchitecture on nitrogen-doped graphene coating Ni foam as flexible battery-type electrodes for advanced supercapattery. Int. J. Hydrogen Energy.

[137] Sainudeen S.S., Joseph A., Joseph M., Sajith V. (2022). Heat transfer phenomena of copper-graphene nanocomposite coated aluminium heat spreaders: An interferometric study. Appl. Therm. Eng..

[138] Sequino L., Sebastianelli G., Vaglieco B.M. (2022). Carbon and Graphene Coatings for the Thermal Management of Sustainable LMP Batteries for Automotive Applications. Materials.

[139] Wang J.X., Mao Y., Miljkovic N. (2024). Nano-Enhanced Graphite/Phase Change Material/Graphene Composite for Sustainable and Efficient Passive Thermal Management. Adv. Sci..

[140] Chen C., Yang Y., Tang X., Qiu R., Wang S., Cao G., Zhang M. (2019). Graphene-Encapsulated FeS_2_ in Carbon Fibers as High Reversible Anodes for Na^+^/K^+^ Batteries in a Wide Temperature Range. Small.

[141] Chen K., Zhang W., Xue L., Chen W., Xiang X., Wan M., Huang Y. (2017). Mechanism of capacity fade in sodium storage and the strategies of improvement for FeS_2_ anode. ACS Appl. Mater. Interfaces.

[142] Gong Y., Sun Y., Li Y., Wu C., Bai Y. (2024). Rational Design of 3D Hierarchical Fe_3_S_4_ for Superior Sodium-Ion Battery Anode Material. Adv. Sustain. Syst..

[143] Hou T., Yue S., Sun X., Fan A., Chen Y., Wang M., Cai S., Zheng C., Liao B., Zhao J. (2020). Nitrogen-Doped graphene coated FeS_2_ microsphere composite as high-performance anode materials for sodium-ion batteries enhanced by the chemical and structural synergistic effect. Appl. Surf. Sci..

[144] Cao J., Wang Y., Li Z., Deng L., Wu K., Wang Y. (2023). Effect of Ti doping on the structure and electrochemical properties of Na_3_V_2_(PO_4_)_2_F_3_ as anode material for sodium ion batteries. Ferroelectrics.

[145] Wang Z.Y., Zheng R., Li W.J., Ma Y.J., Yu K.H., Lv P., Wei W. (2020). Plasma assisted fabrication of a NaTi_2_(PO_4_)_3_@Gr nanocomposite for high-rate and long cycle-life sodium-ion batteries. Sustain. Energy Fuels.

[146] Yuan T., Wang Y., Zhang J., Pu X., Ai X., Chen Z., Yang H., Cao Y. (2019). 3D graphene decorated Na_4_Fe_3_(PO_4_)_2_(P_2_O_7_) microspheres as low-cost and high-performance cathode materials for sodium-ion batteries. Nano Energy.

[147] Dutta P.K., Sen U.K., Mitra S. (2014). Excellent electrochemical performance of tin monosulphide (SnS) as a sodium-ion battery anode. RSC Adv..

[148] Qin J., Shi H., Huang K., Lu P., Wen P., Xing F., Yang B., Ye M., Yu Y., Wu Z.S. (2021). Achieving stable Na metal cycling via polydopamine/multilayer graphene coating of a polypropylene separator. Nat. Commun..

[149] Wang T., Zhang K., Park M., Lau V.W.H., Wang H., Zhang J., Zhang J., Zhao R., Yamauchi Y., Kang Y.M. (2020). Highly Reversible and Rapid Sodium Storage in GeP3 with Synergistic Effect from Outside-In Optimization. ACS Nano.

[150] Liu R., Li Y., Wang C., Xiao N., He L., Guo H., Wan P., Zhou Y., Qiu J. (2018). Enhanced electrochemical performances of coal liquefaction residue derived hard carbon coated by graphene as anode materials for sodium-ion batteries. Fuel Proc. Technol..

[151] Xiong T., Zhang D., Yeo J.Y., Zhan Y., Ong Y.K., Alava Limpo C.M., Shi L., Rao Y., Pu Y., Lai W. (2024). Interfacial design towards stable zinc metal-free zinc-ion batteries with high energy density. J. Mater. Chem. A.

[152] Xia K., Li L., Qiu Y., Weng J., Shen S., Chen M., Zhuang Y., Wen Y., Yang C., Liu Z. (2024). Graphene acid-enhanced interfacial layers with high Zn^2+^ ion selectivity and desolvation capability for corrosion-resistant Zn-metal anodes. J. Mater. Chem. A.

[153] Wang M., Wang Q., Yao H., Su F., Shan Z., Shen H., Liu T., Zhao J., Ding C. (2023). Interfacial regulation and protection by conductive graphene coating induces highly reversible zinc behavior for durable aqueous zinc-ion batteries. J. Alloys Compd..

[154] Etesami M., Khezri R., Motlagh S.R., Gopalakrishnan M., Mano P., Namuangruk S., Wannapaiboon S., Yonezawa T., Somwangthanaroj A., Kheawhom S. (2024). Eco-friendly synthesis of bimetallic FeCo nanocatalysts within heteroatom-doped carbon for oxygen reduction and zinc-air battery enhancement. Mater. Today Energy.

[155] Wang C., Zhao Z., Li X., Yan R., Wang J., Li A., Duan X., Wang J., Liu Y., Wang J. (2017). Three-Dimensional Framework of Graphene Nanomeshes Shell/Co_3_O_4_ Synthesized as Superior Bifunctional Electrocatalyst for Zinc-Air Batteries. ACS Appl. Mater. Interfaces.

[156] Prabakar S.J.R., Park C., Ikhe A.B., Sohn K.S., Pyo M. (2017). Simultaneous Suppression of Metal Corrosion and Electrolyte Decomposition by Graphene Oxide Protective Coating in Magnesium-Ion Batteries: Toward a 4-V-Wide Potential Window. ACS Appl. Mater. Interfaces.

[157] Zhang S., Yu H., Gao Y., Zhu K., Wu H., Cao D. (2023). An organic composite anode with multiple active points for aqueous lithium-ion batteries. Compos. B Eng..

[158] Wang C., Yao Q., Gan Y., Zhang Q., Guan L., Zhao Y. (2022). Monodispersed SWNTs Assembled Coating Layer as an Alternative to Graphene with Enhanced Alkali-ion Storage Performance. Chin. J. Struct. Chem..

[159] Grigoras K., Ahopelto J., Prunnila M., Canham L. (2018). Porous silicon supercapacitors. Handbook of Porous Silicon.

[160] Chatterjee S., Carter R., Oakes L., Erwin W.R., Bardhan R., Pint C.L. (2014). Electrochemical and corrosion stability of nanostructured silicon by graphene coatings: Toward high power porous silicon supercapacitors. J. Phys. Chem. C.

[161] Xiang X., You X., Liu D., Liu X., Yang S., Qiu Y., Tang H., Xie Z., Zheng H., Li J. (2019). A hybrid supercapacitor constructed by graphene wrapped ordered meso-porous Si based electrode. Coll. Surf. A.

[162] Zhong M., Wang X., Huang Y., Li L., Gao S., Tian Y., Shen W., Zhang J., Guo S. (2022). Anthracite-derived carbon-based electrode materials for high performance lithium ion capacitors. Fuel Proc. Technol..

[163] Liu T., Shao G., Ji M. (2014). Electrodeposition of Ni(OH)_2_/Ni/graphene composites under supergravity field for supercapacitor application. Mater. Lett..

[164] Lv J., Liang T., Yang M., Ken S., Hideo M. (2017). Investigation of microstructures of ZnCo_2_O_4_ on bare Ni foam and Ni foam coated with graphene and their supercapacitors performance. J. Energy Chem..

[165] Tong H., Gong D., Liu J., Xiao J., Chen X., Wu Y., Zhou Y., Shen L., Zhang X. (2022). High-performance 2.5 V supercapacitor with high energy density and long cycling stability based on graphene coated oxygen-vacancy birnessite. J. Alloys Compd..

[166] Ning J., Hao L., Zhang X., Liang M., Zhi L. (2014). High-quality graphene grown directly on stainless steel meshes through CVD process for enhanced current collectors of supercapacitors. Sci. China Technol. Sci..

[167] Zhou S., Xie Q., Wu S., Huang X., Zhao P. (2017). Influence of graphene coating on supercapacitive behavior of sandwich-like N- and O-enriched porous carbon/graphene composites in aqueous and organic electrolytes. Ionics.

[168] Xie Q., Zhou S., Wu S., Zhang Y., Zhao P. (2017). Supercapacitive behavior of laminar-structured carbon cloth with alternating graphene and hybrid nanofibers: A synergistic effect of graphene-coating and post-oxidization. Appl. Surf. Sci..

[169] İyidoğan D., Akdemir Ö., Eryilmaz J., Kaplan G., Çavus M.B., Haciismailoğlu B., Alper M., Çobanoğlu Ö. (2019). Projection of sciences onto textile and fashion: Nano-technology and chargeable fabric example—Part II. Tekstil ve Muhendis.

[170] Dericiler K., Hezarkhani M., Tabrizi I.E., Dogan S., Berktas I., Erdem E., Advani S.G., Yildiz M., Sas H.S., Saner Okan B. (2023). Effect of Interleaved Electrolyte Forms on Macro-scaled Structural Hybrid Supercapacitors with Asymmetric Configurations of Graphene-coated Carbon Fabric Electrodes. Appl. Compos. Mater..

[171] Lei C., Markoulidis F., Wilson P., Lekakou C. (2016). Phenolic carbon cloth-based electric double-layer capacitors with conductive interlayers and graphene coating. J. Appl. Electrochem..

[172] Lee G.W., Seol C., Kim K.M., Lim S.T., Shim G.H., Kim S.M., Ahn H.S. (2021). Thermally annealed self-assembled three-dimensional graphene for direct construction of porous flow distributor in polymer electrolyte membrane fuel cell. Int. J. Hydrogen Energy.

[173] Arya A.K., Singh Raman R.K., Parmar R., Amati M., Gregoratti L., Saxena S. (2024). Graphene-Coated Ni-Cu Alloys for Durable Degradation Resistance of Bi-Polar Plates for Proton Exchange Membrane Fuel Cells: Remarkable Role of Alloy Composition. Small.

[174] Sun X.W., Li X.C., Zhao J.X., Wang J.H., Liu F., Zhao P.W., Dai Z.Q., Zheng L.L. (2023). Research Progress on Protective Coatings for Bipolar Plates of Proton Exchange Membrane Fuel Cells. Surf. Technol..

[175] Yu F., Wang K., Cui L., Wang S., Hou M., Xiong F., Zou R., Gao P., Peng H., Liu Z. (2022). Vertical-Graphene-Reinforced Titanium Alloy Bipolar Plates in Fuel Cells. Adv. Mater..

[176] Okonkwo P.C., Emori W., Uzoma P.C., Mansir I.B., Radwan A.B., Ige O.O., Abdullah A.M. (2022). A review of bipolar plates materials and graphene coating degradation mechanism in proton exchange membrane fuel cell. Int. J. Energy Res..

[177] Ren Y.J., Anisur M.R., Qiu W., He J.J., Al-Saadi S., Singh R.K. (2017). Raman Degradation of graphene coated copper in simulated proton exchange membrane fuel cell environment: Electrochemical impedance spectroscopy study. J. Power Sources.

[178] Nam H.N., Phung Q.M., Choeichom P., Yamauchi Y., Saito N. (2024). First-principles studies of enhanced oxygen reduction reactions on graphene- and nitrogen-doped graphene-coated platinum surfaces. Phys. Chem. Chem. Phys..

[179] Hsu W.H., Tsai H.Y., Huang Y.C. (2017). Characteristics of Carbon Nanotubes/Graphene Coatings on Stainless Steel Meshes Used as Electrodes for Air-Cathode Microbial Fuel Cells. J. Nanomater..

[180] Song T.S., Fei K., Zhang H., Yuan H., Yang Y., Ouyang P., Xie J. (2018). High efficiency microbial electrosynthesis of acetate from carbon dioxide using a novel graphene-nickel foam as cathode. J. Chem. Technol. Biotechnol..

[181] Holze R., Breitkopf C., Swider-Lyons K. (2016). Kinetics of Fast Redox Systems for Energy Storage. Springer Handbook of Electrochemical Energy.

[182] Wu Y., Holze R. (2018). Electrocatalysis at Electrodes for Vanadium Redox Flow Batteries. Batteries.

[183] Xie X., Holze R. (2022). Electrode Kinetic Data: Geometric vs. Real Surface Area. Batteries.

[184] Pahlevaninezhad M., Miller E.E., Yang L., Prophet L.S., Singh A., Storwick T., Pahlevani M., Pope M.A., Roberts E.P.L. (2023). Exfoliated Graphene Composite Membrane for the All-Vanadium Redox Flow Battery. ACS Appl. Energy Mater..

[185] Roth C., Noack J., Skyllas-Kazacos M. (2023). Flow Batteries.

[186] Lee D., Roh J.S., Hwang I., Jung Y., Lee H., Ock I.W., Kim S., Sun S., Yang S., Park H.B. (2022). Multilayered Graphene-Coated Metal Current Collectors with High Electrical Conductivity and Corrosion Resistivity for Flow-Electrode Capacitive Mixing. ACS Sustain. Chem. Eng..

